# How Criterion Scores Predict the Overall Impact Score and Funding Outcomes for National Institutes of Health Peer-Reviewed Applications

**DOI:** 10.1371/journal.pone.0155060

**Published:** 2016-06-01

**Authors:** Matthew K. Eblen, Robin M. Wagner, Deepshikha RoyChowdhury, Katherine C. Patel, Katrina Pearson

**Affiliations:** Office of Extramural Research, National Institutes of Health, Bethesda, Maryland, United States of America; Charles P. Darby Children's Research Institute, 173 Ashley Avenue, Charleston, SC 29425, USA, UNITED STATES

## Abstract

Understanding the factors associated with successful funding outcomes of research project grant (R01) applications is critical for the biomedical research community. R01 applications are evaluated through the National Institutes of Health (NIH) peer review system, where peer reviewers are asked to evaluate and assign scores to five research criteria when assessing an application’s scientific and technical merit. This study examined the relationship of the five research criterion scores to the Overall Impact score and the likelihood of being funded for over 123,700 competing R01 applications for fiscal years 2010 through 2013. The relationships of other application and applicant characteristics, including demographics, to scoring and funding outcomes were studied as well. The analyses showed that the Approach and, to a lesser extent, the Significance criterion scores were the main predictors of an R01 application’s Overall Impact score and its likelihood of being funded. Applicants might consider these findings when submitting future R01 applications to NIH.

## Introduction

The National Institutes of Health (NIH) is the world's leading biomedical and behavioral research organization and spends about three-quarters of its nearly $30.1 billion budget on extramural grant research funding to support research in universities, medical schools and research institutions [1]. Peer review is the cornerstone of the NIH’s extramural research program. Applications for research funding from NIH’s extramural research program are vetted through the peer review process [2]. Over the years, the NIH has made periodic efforts to improve its peer review system to ensure fairness and efficiency in evaluating grant applications. The most recent effort began in June of 2007 [3]. The enhancements to the NIH peer review system were implemented, in phases, beginning in 2009 [4]. The key modifications included changes to the grant application review criteria, quantitative scoring of five distinct review criteria (criterion scores), implementation of a new 1–9 point scoring system for both the review criteria and the application as a whole (the “Overall Impact” score), and the clustering of applications for the peer review of new and early stage investigator (ESI) applications for R01s, NIH’s major research grant activity code (see Career Stage of Investigators definition in Table 1). Also, as part of this enhancement, the NIH committed itself to continuous monitoring and evaluation of the peer review system.

**Table 1:** Summary Statistics for R01-Equivalent Applications, FY 2010–2013.

	No. (%) of Discussed Applications	Overall Impact^a^ Mean (st. dev)	Approach Mean (st. dev)	Significance Mean(st. dev)	Innovation Mean (st. dev)	Investigator(s) Mean (st. dev)	Environment Mean (st. dev)	No. (%) of Funded Applications
**Application/Applicant Characteristic**^b^	** **	** **	** **	** **	** **	** **	** **	** **
**(n = total applications, discussed and non-discussed)**								
**Application Type**^c^	** **	** **	** **	** **	** **	** **	** **	** **
New (Type 1) (n = 100,104)	54,415 (54.4)	37.1 (13.1)	4.4 (1.4)	3.2 (1.2)	3.2 (1.1)	2.4 (1)	2.1 (0.9)	14,213 (14.2)
Renewal (Type 2) (n = 22,714)	16,559 (72.9)	30.9 (12.2)	3.6 (1.2)	2.6 (1)	2.8 (1)	1.8 (0.8)	1.7 (0.6)	6,847 (30.1)
Revision (Type 3) (n = 568)	440 (77.5)	36.3 (13.8)	3.8 (1.3)	2.9 (1.1)	3 (1.1)	1.9 (0.7)	1.7 (0.6)	151 (26.6)
Change of Awarding IC Renewal (Type 9) (n = 321)	237 (73.8)	29.7 (12.4)	3.5 (1.3)	2.6 (1.1)	2.7 (1)	1.8 (0.8)	1.7 (0.6)	115 (35.8)
**Application Submission Number**^d^								
Initial submission (A0) (n = 86,375)	43,967 (50.9)	38.1 (12.9)	4.5 (1.4)	3.2 (1.2)	3.3 (1.2)	2.4 (1)	2.1 (0.9)	9,693 (11.2)
First Resubmission (A1) (n = 32,320)	23,781 (73.6)	31.7 (12.5)	3.7 (1.3)	2.7 (1)	2.8 (1)	2 (0.9)	1.8 (0.7)	9,891 (30.6)
Second resubmission (A2) (n = 5,012)	3,903 (77.9)	32.2 (12.8)	3.7 (1.3)	2.8 (1)	3 (1)	2.2 (0.9)	2 (0.7)	1,742 (34.8)
**Career Stage of Investigators**^e^								
Experienced Investigator (n = 84,647)	49,802 (58.8)	33.9 (12.6)	4 (1.3)	2.9 (1.1)	3 (1.1)	2 (0.9)	1.9 (0.7)	15,899 (18.8)
Early Stage Investigator (ESI) (n = 18,318)	11,243 (61.4)	38.4 (13.3)	4.5 (1.4)	3.2 (1.2)	3.3 (1.1)	2.6 (1)	2.2 (0.9)	3,222 (17.6)
Non-ESI New Investigator (n = 20,742)	10,606 (51.1)	41.1 (13.7)	4.9 (1.4)	3.5 (1.2)	3.6 (1.2)	2.9 (1.2)	2.4 (1.1)	2,205 (10.6)
**Multiple Principal Investigator (MPI) Status**^f^								
Single PI Application (n = 105,235)	61,213 (58.2)	35.6 (13.2)	4.2 (1.4)	3.1 (1.2)	3.2 (1.1)	2.3 (1)	2 (0.9)	18,531 (17.6)
MPI Application (n = 18,472)	10,438 (56.5)	36.1 (13)	4.2 (1.4)	3 (1.1)	3.1 (1.1)	2.2 (1)	1.9 (0.8)	2,795 (15.1)
**Human and/or Animal Subject Involvement**^g^								
No Human or Animal Subjects (n = 21,532)	12,391 (57.5)	34.4 (13.2)	4.1 (1.5)	3.1 (1.2)	3.3 (1.2)	2.3 (1)	2.1 (0.9)	4,295 (19.9)
Animals Subjects Only (n = 55,055)	31,799 (57.8)	35.5 (13.1)	4.3 (1.4)	3 (1.1)	3.1 (1.1)	2.3 (1)	2 (0.8)	9,836 (17.9)
Humans Subjects Only (n = 36,011)	20,990 (58.3)	36.5 (13.3)	4.3 (1.4)	3.1 (1.2)	3.2 (1.2)	2.2 (1)	1.9 (0.9)	5,557 (15.4)
Human and Animal Subjects (n = 11,109)	6,471 (58.3)	36.2 (12.8)	4.3 (1.3)	3 (1.1)	3 (1)	2.3 (0.9)	2 (0.8)	1,638 (14.7)
**NIH Research Grant Funding Rank of Institution**^h^								
Rank 1–30 (n = 44,218)	28,090 (63.5)	34.6 (12.9)	4 (1.3)	2.9 (1.1)	3 (1.1)	2.1 (0.9)	1.8 (0.7)	8,960 (20.3)
Rank 31–100 (n = 42,276)	24,485 (57.9)	35.7 (13.1)	4.2 (1.4)	3.1 (1.1)	3.1 (1.1)	2.3 (0.9)	2 (0.8)	7,193 (17.0)
Rank 101–200 (n = 19,711)	10,752 (54.5)	36.2 (13.2)	4.3 (1.4)	3.2 (1.2)	3.2 (1.1)	2.4 (1)	2.1 (0.9)	3,104 (15.7)
Rank > 200 (n = 16,300)	7,936 (48.7)	37.9 (13.6)	4.6 (1.4)	3.3 (1.2)	3.4 (1.2)	2.6 (1.1)	2.4 (1)	1,991 (12.2)
No Previous Funding (n = 1,202)	388 (32.3)	44.6 (16.4)	5.8 (1.8)	4.3 (1.7)	4.5 (1.8)	4 (2)	3.8 (2)	78 (6.5)
**Institution Type**^i^								
Medical School (n = 64,270)	38,012 (59.1)	35.3 (13)	4.2 (1.4)	3 (1.1)	3.1 (1.1)	2.2 (0.9)	2 (0.8)	11,564 (18.0)
Higher Education (excl. Medical) (n = 36,821)	20,549 (55.8)	36.1 (13.4)	4.3 (1.4)	3.2 (1.2)	3.2 (1.2)	2.3 (1)	2.1 (0.9)	5,980 (16.2)
Independent Hospital (n = 9,214)	5,604 (60.8)	35.6 (13)	4.2 (1.4)	3 (1.1)	3 (1.1)	2.2 (0.9)	1.8 (0.8)	1,658 (18.0)
Research Institute (n = 9,716)	5,627 (57.9)	35.7 (13.1)	4.2 (1.4)	3 (1.1)	3.1 (1.1)	2.3 (1)	2 (0.8)	1,636 (16.8)
Other Institution (n = 3,686)	1,859 (50.4)	37.5 (14.1)	4.7 (1.6)	3.4 (1.4)	3.5 (1.5)	2.7 (1.5)	2.5 (1.6)	488 (13.2)
**Race**^j^								
White (n = 76,924)	46,614 (60.6)	34.8 (13)	4.1 (1.4)	3 (1.1)	3.1 (1.1)	2.2 (0.9)	1.9 (0.8)	14,652 (19.0)
Asian (n = 24,316)	13,329 (54.8)	36.6 (13.1)	4.4 (1.4)	3.2 (1.1)	3.3 (1.1)	2.4 (1)	2.1 (0.8)	3,745 (15.4)
Black (n = 1,596)	735 (46.1)	38.1 (13.6)	4.8 (1.5)	3.4 (1.3)	3.5 (1.3)	2.7 (1.2)	2.3 (1.1)	188 (11.8)
Other (n = 10,014)	5,364 (53.6)	36.7 (13.1)	4.3 (1.4)	3.1 (1.1)	3.2 (1.1)	2.3 (1)	2 (0.9)	1,404 (14.0)
Unknown (n = 7,285)	3,541 (48.6)	40.1 (13.9)	4.8 (1.5)	3.5 (1.3)	3.6 (1.3)	2.8 (1.3)	2.4 (1.2)	751 (10.3)
Withheld (n = 3,572)	2,068 (57.9)	36.5 (13.8)	4.3 (1.4)	3.1 (1.1)	3.2 (1.1)	2.3 (1)	2 (0.8)	586 (16.4)
**Ethnicity**^k^								
Non-Hispanic (n = 84,563)	50,260 (59.4)	35.2 (13)	4.2 (1.4)	3 (1.1)	3.1 (1.1)	2.2 (0.9)	2 (0.8)	15,486 (18.3)
Hispanic (n = 3,903)	2,194 (56.2)	36.1 (13.1)	4.4 (1.4)	3.1 (1.2)	3.2 (1.1)	2.4 (1)	2.1 (0.9)	648 (16.6)
MPI Multiple Ethnicities (n = 8,977)	4,971 (55.4)	36.5 (13)	4.3 (1.4)	3.1 (1.1)	3.1 (1.1)	2.2 (1)	2 (0.8)	1,311 (14.6)
Unknown (n = 22,514)	12,032 (53.4)	36.9 (13.6)	4.5 (1.5)	3.2 (1.2)	3.3 (1.2)	2.5 (1.1)	2.2 (1)	3,207 (14.2)
Withheld (n = 3,750)	2,194 (58.5)	35.7 (13.4)	4.2 (1.4)	3.1 (1.2)	3.1 (1.1)	2.3 (1)	2 (0.8)	674 (18.0)
**Gender**^l^								
Male (n = 82,257)	48,104 (58.5)	35.3 (13.2)	4.2 (1.4)	3 (1.1)	3.1 (1.1)	2.2 (1)	2 (0.8)	14,764 (17.9)
Female (n = 31,667)	18,269 (57.7)	36.2 (13)	4.3 (1.4)	3.1 (1.1)	3.2 (1.1)	2.3 (1)	2 (0.9)	5,184 (16.4)
MPI Multiple Gender (n = 8,357)	4,614 (55.2)	36.2 (13)	4.3 (1.4)	3.1 (1.1)	3.1 (1.1)	2.2 (1)	2 (0.8)	1,230 (14.7)
Unknown (n = 530)	202 (38.1)	41.8 (14.6)	5.4 (1.7)	3.9 (1.6)	4.1 (1.6)	3.4 (1.7)	3 (1.7)	44 (8.3)
Withheld (n = 896)	462 (51.6)	39.5 (14.1)	4.7 (1.5)	3.4 (1.3)	3.5 (1.3)	2.8 (1.3)	2.4 (1.2)	104 (11.6)
**Degree**^m^								
PhD (n = 84,297)	48,385 (57.4)	35.5 (13.1)	4.2 (1.4)	3.1 (1.1)	3.2 (1.1)	2.3 (1)	2 (0.8)	14,696 (17.4)
MD-PhD (n = 13,368)	7,948 (59.5)	36 (13.2)	4.3 (1.4)	3 (1.1)	3.1 (1.1)	2.3 (0.9)	2 (0.8)	2,371 (17.7)
MD (n = 15,929)	9,935 (62.4)	35.3 (13.1)	4.2 (1.4)	2.9 (1.1)	3.1 (1.1)	2.1 (1)	1.9 (0.8)	2,972 (18.7)
MPI Multiple Degree Types (n = 8,695)	4,892 (56.3)	36.6 (12.9)	4.3 (1.4)	3 (1.1)	3.1 (1.1)	2.2 (1)	1.9 (0.8)	1,237 (14.2)
Other (n = 1,418)	491 (34.6)	44.9 (14)	5.5 (1.6)	4 (1.5)	4.2 (1.6)	3.5 (1.7)	3 (1.7)	50 (3.5)
**Age Group (Years)**^n^								
24–35 (n = 3,159)	1,878 (59.4)	37.4 (12.7)	4.4 (1.4)	3.2 (1.2)	3.3 (1.2)	2.6 (1)	2.1 (1)	559 (17.7)
36–45 (n = 31,995)	19,185 (60.0)	36.7 (13.1)	4.3 (1.3)	3.1 (1.1)	3.2 (1.1)	2.4 (1)	2.1 (0.8)	5,646 (17.6)
46–55 (n = 36,695)	21,318 (58.1)	35.2 (13.2)	4.2 (1.4)	3 (1.1)	3.1 (1.1)	2.3 (1)	2 (0.8)	6,476 (17.6)
56–65 (n = 21,635)	12,607 (58.3)	34.1 (13.1)	4.1 (1.4)	3 (1.2)	3.1 (1.1)	2.1 (1)	1.9 (0.8)	4,033 (18.6)
65+ (n = 6,446)	3,529 (54.7)	34.1 (13.4)	4.2 (1.5)	3 (1.2)	3.2 (1.2)	1.9 (0.9)	1.9 (0.8)	1,123 (17.4)
MPI Multiple Age Groups (n = 13,822)	7,782 (56.3)	36.2 (12.9)	4.2 (1.4)	3 (1.1)	3.1 (1.1)	2.2 (1)	1.9 (0.8)	2,062 (14.9)
Unknown (n = 9,955)	5,352 (53.8)	36.8 (13.3)	4.4 (1.5)	3.2 (1.2)	3.3 (1.2)	2.4 (1.1)	2.2 (1)	1,427 (14.3)
**Fiscal Year of Application**								
2010 (n = 30,487)	18,243 (59.8)	37.1 (14.1)	4.4 (1.5)	3.3 (1.2)	3.5 (1.2)	2.5 (1.1)	2.3 (1)	5,999 (19.7)
2011 (n = 31,216)	18,177 (58.2)	35.9 (13.2)	4.3 (1.4)	3.1 (1.1)	3.2 (1.1)	2.3 (1)	2 (0.8)	5,237 (16.8)
2012 (n = 31,709)	18,065 (57.0)	34.4 (12.6)	4.1 (1.3)	2.9 (1.1)	3 (1.1)	2.1 (0.9)	1.9 (0.8)	5,348 (16.9)
2013 (n = 30,295)	17,166 (56.7)	35.1 (12.5)	4.2 (1.3)	2.9 (1.1)	3 (1)	2.1 (0.9)	1.8 (0.7)	4,742 (15.7)
**Total (n = 123,707)**	**71,651 (57.9)**	**35.6 (13.2)**	**4.3 (1.4)**	**3.1 (1.2)**	**3.2 (1.1)**	**2.3 (1)**	**2 (0.9)**	**21,326 (17.2)**

^a^ Overall Impact score averages only include discussed applications.

^b^ Other application and applicant characteristics evaluated, but not shown here due to space limitations, are: Council round of review, human or animal subject concerns, solicitation type (unsolicited, program announcement or request for application), locus of review (Center for Scientific Review v. other NIH Institutes and Centers), review group type (standing study section v. special emphasis panel), direct costs requested, # of years of support requested, the NIH administering Institute or Center (IC), the geographical region of the institution and the previous NIH funding history of the applicant.

^c^ A new application is a type 1 application. A type 2 application is a renewal, also known as competing continuation. A type 3 application can be a competing revision for additional support to expand the scope of study or can be a non-competing administrative supplement application for additional support to cover increased costs. A type 9 application is a renewal for which the awarding institute or center changes.

^d^ An application submitted for the first time is an A0 application or an initial submission. A previously submitted unfunded A0 application resubmitted for new funding consideration is an A1 application or a first resubmission. A previously unfunded A1 application resubmitted for new funding consideration is an A2 application or a second resubmission. The policy on resubmission in place for applications submitted during the study period, FY 2010-FY 2013, can be found at http://grants.nih.gov/grants/guide/notice-files/NOT-OD-09-003.html.

^e^ A new investigator is defined as a principal investigator who has not previously competed successfully as a principal investigator for a substantial independent research award. A new investigator who is within 10 years of completing his/her terminal research degree or is within 10 years of completing medical residency (or equivalent) is considered an early stage investigator. A principal investigator who is not a new investigator is an experienced investigator. A list of NIH grant activities that do not disqualify a principal investigator from being considered as a new investigator can be found at http://grants.nih.gov/grants/new_investigators/index.htm.

^f^ An application including only one principal investigator (PI) is a single PI application. An application including more than one principal investigator is a multiple PI (MPI) application.

^g^ An application involving (1) only human subjects for research is a humans only application, (2) only animal subjects for research is an animals only application, (3) both human and animal subjects for research is a humans and animals application, and (4) neither human nor animal subjects for research is a no humans or animals application.

^h^ An application's rank is based on the rank order of the application's submitting organization or institution with respect to the total amount of NIH research grant funding received by that organization compared to all other organizations over the five year period prior to the fiscal year of the application. The lower the rank, the higher is the previous level of funding from NIH.

^i^ The type of the institution or organization submitting the application.

^j^ Race of a principal investigator is the racial category that was self-reported by the principal investigator. Applications whose principal investigator reports more than one race category or applications with multiple principal investigators who report different race categories are included in the 'Other' category.

^k^ Ethnicity of a principal investigator is the ethnicity selection that was self-reported by the principal investigator. Applications with multiple principal investigators who report different ethnicities are included in the 'MPI Multiple Ethnicities' category.

^l^ Gender of a principal investigator is the gender selection that was self-reported by the principal investigator. Applications with multiple principal investigators who report different genders are included in the 'MPI Multiple Gender' category.

^m^ Degree represents the highest degree attained by a principal investigator. Applications with multiple principal investigators reporting more than one degree type are included in the 'MPI Multiple Degree Types' category. The "Other" degree category includes degree types such as veterinary, dental and unknown degrees.

^n^ Age of a principal investigator is calculated by subtracting the principal investigator's birth year from the application's fiscal year. Applications with multiple principal investigators who report different age group categories are included in the ‘MPI Multiple Age Groups’ category. Those with an erroneous birth date (less than 24 or greater than 90) or missing birth date are included in the 'Unknown' age category.

NIH peer review is a two-stage process. In the first level of review, research grant applications are evaluated for scientific and technical merit by a Scientific Review Group (SRG), also known as a study section, comprised primarily of non-federal scientists with expertise in relevant scientific disciplines and current research areas. Reviewers from the SRG consider five criteria when assessing an application’s scientific and technical merit. The criteria for research grants are Significance, Investigator(s), Innovation, Approach, and Environment. Additional criteria, such as whether an application involves human or animal subjects, or is a renewal, revision or resubmission, are considered when applicable (see http://grants.nih.gov/grants/peer_review_process.htm for a full description of the criteria). The more meritorious applications are discussed in full at SRG meetings where a final Overall Impact score is assigned by each reviewer. The final Overall Impact score of each discussed application is the mean of all eligible reviewers’ Impact scores times 10. Thus, the final Overall Impact scores range from 10 (high impact) through 90 (low impact). Applications that are not discussed (ND) do not receive a final numerical Overall Impact score.

The second level of peer review is performed by Advisory Councils/Boards for each NIH Institute and Center (IC). This second level of review assesses the relevance of the application’s proposed research to the IC’s programs and priorities, resulting in recommendations for funding. Based on these recommendations, as well as input from NIH program staff and considering the mission and goals of their respective IC, the IC directors make the final funding decisions.

The introduction of quantitative scores for the five research review criteria, beginning in fiscal year (FY) 2010, enabled the examination of the relationship of these criteria to first level peer review outcomes, i.e., the Overall Impact score, and to the likelihood of being funded.

Previous studies of the scientific research peer review process at NIH and other funding agencies have evaluated how the characteristics of peer reviewers, the peer review process, grant applicants and their institutions, and research topics are associated with peer review outcomes [5–15]. Lindner et al. examined how the variation in Overall Impact scores was explained by the criterion scores and concluded that all the criteria were important contributors to the Overall Impact score [15]. What distinguishes this work from earlier studies is that multivariate techniques were used to estimate the magnitude of the relationship between each individual criterion score and the Overall Impact score. Furthermore, the analysis was broadened to include the relationship between the criterion scores and funding outcomes. This study also measured the degree to which additional factors, including the application’s administrative characteristics, the demographics of the applicant, and characteristics of the applicant’s institution, were associated with peer review and funding outcomes after adjusting for application-specific ratings of scientific and technical merit, as embodied in the criterion scores.

## Methods

Data from 123,707 competing R01-equivalent applications (R01s and R37s) submitted to NIH during fiscal year (FY) 2010 to FY 2013 and peer reviewed were included in the current analysis. These data were extracted from the Information for Management, Planning, Analysis, and Coordination II (IMPACII), the database of record for information collected from NIH extramural grant applications, awards and applicants during the receipt, review and award management process. For each application, data were obtained on whether the application was funded, its final Overall Impact score, and its five research criterion scores, which were delinked from the reviewers providing the scores. The research criterion scores were calculated for each criterion by averaging all individual criterion scores available for a particular application. In addition, data were extracted on other characteristics related to the application (such as whether it was a new or renewal application), the applicant (such as applicant demographics and personal NIH funding history) and the applicant’s institution (such as the institutional funding history with NIH). All demographic data were self-reported, on a voluntary basis, by the applicants. Data on the SRG where the application was reviewed were also obtained. See Table 1 for a full list of variables evaluated for each application. Descriptive summary statistics, as well as correlations between the five criterion scores and the Overall Impact score were produced.

### Models

Two general models were developed: 1) the Impact model, a linear regression model with the Overall Impact score serving as the dependent variable; and 2) the Funding model, a logistic regression model with the likelihood of being funded serving as the dependent variable. The five research criteria were used as the main predictors in both models, controlling for other application and applicant characteristics delineated in Table 1. Both models controlled for the FY of the application to account for changes in the distribution of Overall Impact scores or funding patterns over time. Hierarchical random effects models, with applications clustered by SRG, were employed to account for possible differences in scoring behavior and funding outcomes between peer review groups. In addition to controlling for the potential clustering of scores by SRG, the use of random effects, by way of intraclass correlations, allowed for the decomposition of the total variation in the models into two categories: within-SRG variation and between-SRG variation [16–18].

Three sub-models were developed in a step-wise fashion to assess the marginal contribution of each set of characteristics in both general models. Sub-model A focused on the five research criterion scores, including any significant interactions between them. Sub-model B added the other control variables to sub-model A. Sub-model C was identical to sub-model B, but removed the criterion scores. Sub-model C served to illustrate how the various application and applicant characteristics appeared to be associated with the Impact score and relative odds of funding when the quality of the application, as measured by the criterion scores, was not taken into account.

Because the ND applications are not assigned Overall Impact scores, only the 71,651 applications that were discussed in SRG meetings and assigned Overall Impact scores from FY 2010 to FY 2013, were used to fit the Impact model. ND applications were not removed from the Funding model because their funding outcomes were known, and data on the five research criterion scores were still available. However, applications precluded from being considered for funding were removed, i.e., those with unresolved human subject or animal concerns and resubmitted applications that had a previous version funded. Removing these applications left 111,533 R01-equivalent applications for the Funding model.

Data analyses were performed using Stata 13 (StataCorp). Model estimates and their 95% confidence intervals (CIs) were computed. The Funding model results were expressed as odds ratios. For ease of interpretation, the coefficients of the criterion score estimates were inverted in the Funding model, so that odds ratios greater than unity should be interpreted as the magnitude of the increase in odds of funding due to a one unit decrease (improvement) of the given criterion. Results were considered statistically significant if they had a P-value of less than 0.05, using 2-sided testing.

The NIH Office of Human Subjects Research Protections was consulted and determined this work to be classified as a program evaluation that did not require human subjects research review by an Institutional Review Board.

## Results

**Fig 1:**
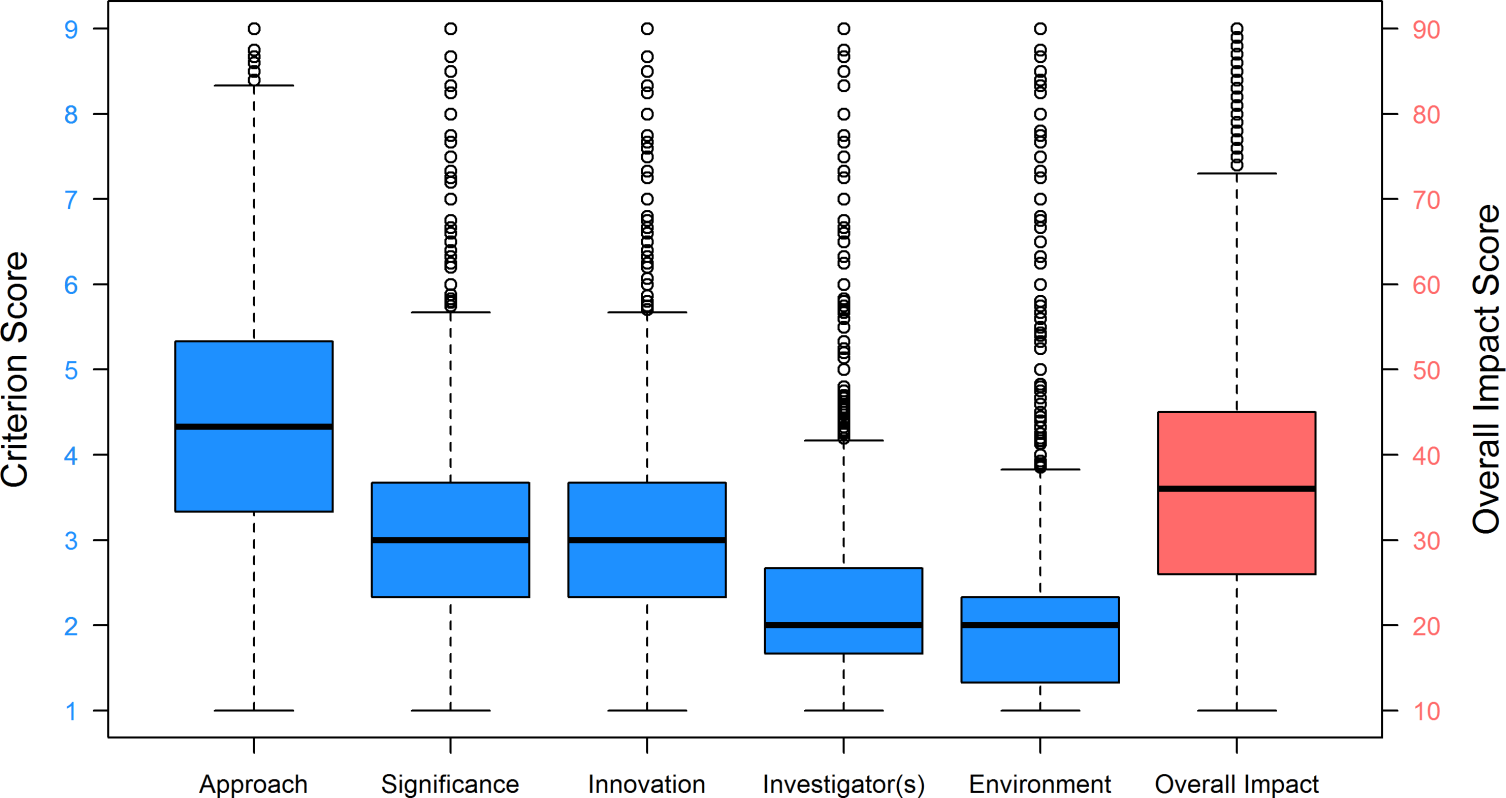
Box Plot Distributions of Criterion and Overall Impact Scores for R01 Applications, FY 2010–2013. Fig 1 shows the box plot distributions of the five research criterion scores (scale: 1–9) and the Overall Impact score (scale: 10–90). Box plot whiskers extend to the most extreme data point which is no more than 1.5 times the interquartile range from the box. Each criterion score N = 123,707 applications; Overall Impact score N = 71,651 applications.

Fig 1 shows the distribution of the Overall Impact score and criterion scores in the form of boxplots. The criterion scores for Approach had the greatest variability and highest (or worst) scores, with an interquartile range (IQR) of 2.0 and median of 4.3. The criterion scores for Significance and Innovation both had IQRs of 1.2 and medians of 3.0. Investigator(s) and Environment criterion scores were clustered in the low score ranges with median scores of 2.0 and IQRs of 1.0, indicating that most applications received excellent marks for Investigator and Environment. Table 2 provides the correlations between the criterion scores for each of the five research criteria and the Overall Impact score. All criteria had moderate to high correlations with one another, ranging from 0.55 between Significance and Environment to 0.75 between Investigator(s) and Environment. Environment had the lowest correlation with the Overall Impact score, whereas Approach had the highest correlation with the Overall Impact score (0.44 and 0.84, respectively).

**Table 2:** Pearson Correlation Matrix of the 5 Research Criteria and Overall Impact Scores.

	Overall Impact	Approach	Significance	Innovation	Investigator	Environment
**Variable**						
Overall Impact	1	-	-	-	-	-
Approach	0.84	1	-	-	-	-
Significance	0.68	0.72	1	-	-	-
Innovation	0.61	0.68	0.72	1	-	-
Investigator(s)	0.53	0.66	0.6	0.6	1	-
Environment	0.44	0.57	0.55	0.56	0.75	1

Table 1 shows that the average Overall Impact scores and funding rates varied widely according to different application characteristics. For example, new (type 1) applications had an average Overall Impact score of 37.1 and funding rate of 14.2% while renewal (type 2) applications fared better, with an average Overall Impact score of 30.9 and funding rate of 30.1%. Initial submissions (A0s) had an average Overall Impact score of 38.1 and funding rate of 11.2%, whereas resubmissions (A1s) had a more favorable average Overall Impact score and funding rate (31.7 and 30.6%, respectively). Applications from Early Stage Investigators (ESIs) had an average Overall Impact score of 38.4 and a 17.6% funding rate, whereas applications from experienced investigators had a better average Overall Impact score and funding rate (33.9 and 18.8%, respectively). Applications submitted by white principal investigators (PIs) had an average Overall Impact score of 34.8 and a funding rate of 19.0%; in contrast, applications submitted by black PIs had poorer outcomes (average Impact score: 38.1; funding rate: 11.8%). Male PIs had Overall Impact scores and funding rates of 35.3 and 17.9%, respectively, whereas female PIs had corresponding worse scores and funding rates of 36.2 and 16.4%, respectively.

**Fig 2:**
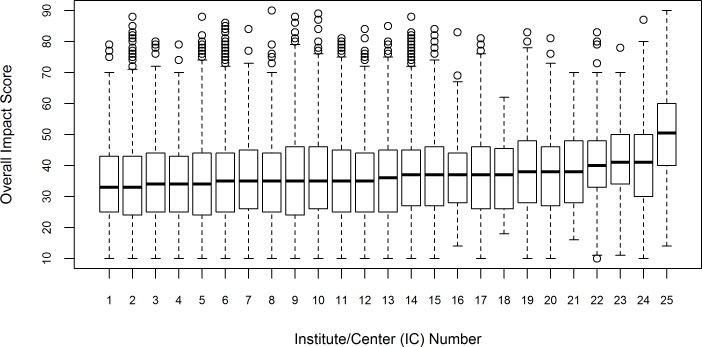
Box Plot Distributions of Overall Impact Scores for R01 Applications by IC, FY 2010–2013. Fig 2 shows the box plot distributions of the Overall Impact score (scale: 10–90) by IC. Box plot whiskers extend to the most extreme data point which is no more than 1.5 times the interquartile range from the box. IC names have been masked. N = 71,651 applications (discussed applications only).

**Fig 3:**
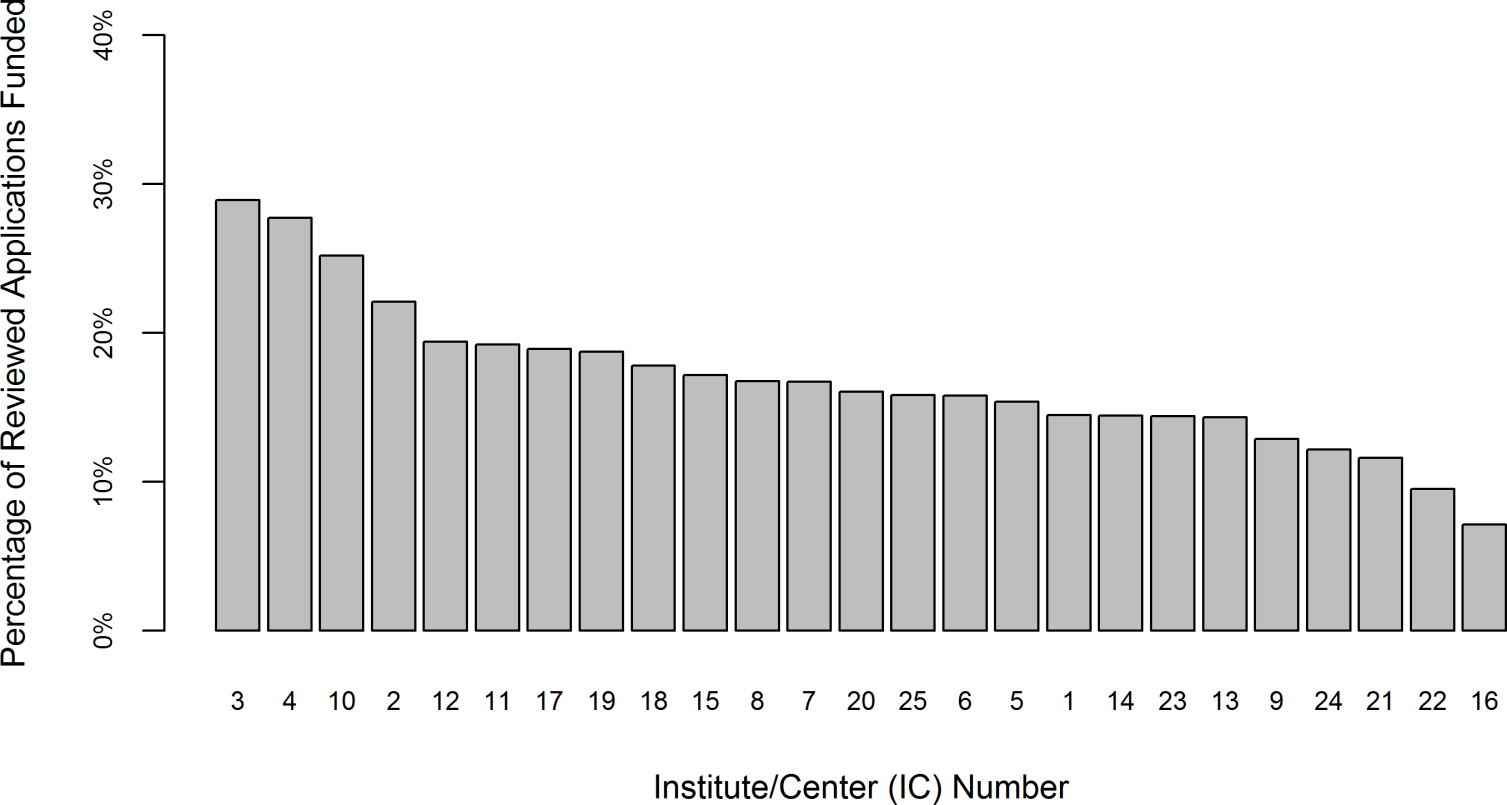
Distributions of Funding Rate for R01 Applications by IC, FY 2010–2013. Fig 3 shows the distribution of the percentage of reviewed applications funded by each IC. IC names have been masked and have been labeled to agree with Fig 3, i.e., the IC labeled as “1” in Fig 2 is the same IC labeled as “1” in Fig 2. N = 123,707.

Fig 2 shows boxplot distributions of the Overall Impact score by IC, with IC names masked. Median scores ranged considerably by IC, from 33 to 50.5. IQRs ranged from 15 to 22 across ICs. Fig 3 shows the percentage of reviewed applications that were funded by each IC. This rate ranged widely from 7.1% to 28.9%. The rank order of the Overall Impact scores and funding rates by ICs, shown in Figs 2 and 3, respectively, do not match as might be expected: ICs that had better (lower) ranges of Overall Impact scores did not necessarily have higher funding levels. This is due, in part, to differences in the number of applications received and available grant funding dollars between the different ICs, and demonstrates the importance of controlling for IC, particularly in the Funding model.

S1 and S2 Tables are similar to Table 1, except that they show summary statistics for discussed and ND applications, respectively. In comparing the two tables, ND applications had worse (higher) mean criterion scores for all five research criteria, compared to discussed applications. Furthermore, the Approach criterion had the worst mean scores for both discussed and ND applications. Among discussed applications, the Approach criterion was more variable, with a higher standard deviation than the other criterion scores, underscoring the former criterion’s importance in predicting the Overall Impact score amongst discussed applications. In contrast to discussed applications, which had an overall 29.8% funding rate over the study period, ND applications had almost no chance of being funded (only one ND application was funded in FY 2010–2013).

The Impact model and Funding model results are shown in Tables 3 and 4, separated by sub-model. In sub-model A, with independent variables limited to the criterion scores, all were highly significant in the Impact model, with the coefficients in rank order for Approach, Significance, Innovation, Investigator(s) and Environment estimated at 7.6 (95% CI, 7.5–7.7), 3.4 (3.3–3.5), 1.4 (1.3–1.5), 1.0 (0.9–1.0) and -0.2 (-0.3–-0.1), respectively. That is, a one point improvement in the Approach score was associated with a 7.6 point improvement in the Overall Impact score, controlling for the other criterion scores. The Funding model results for sub-model A had coefficients in the same rank order, with odds ratio estimates of 6.2 (5.9–6.5), 2.1 (2.0–2.2), 1.5 (1.4–1.6), 1.0 (1.0–1.1) and 0.9 (0.8–0.9), respectively, e.g., for every one point improvement in the Approach score, the odds of funding increased by a factor of 6.2. There was a highly significant interaction between Approach and Significance in both the Impact and Funding models; applications that had good scores on both criteria had better than expected outcomes than would be predicted by their independent effects. Sub-model A explained 74.8% of the variation in Overall Impact scores. This result is similar to the Lindner et al. (*15*) figure of 77.7%. Sub-model A also correctly predicted the funding outcomes of 66.0% of funded applications and 94.7% of unfunded applications, for an overall correct prediction rate of 89.3%. The intraclass correlation coefficient, which measures the amount of variation accounted for by SRGs, was 4.2% in the Impact model and 17.8% in the Funding model; i.e., an application’s criterion scores were much better indicators of its review and funding outcomes than the SRG in which it was reviewed.

**Table 3:** Impact Score Model^a^ Results for R01-Equivalent Applications, FY 2010–2013.

	Sub-Model A		Sub-Model B		Sub-Model C	
	Estimate (95% CI)	P Value	Estimate (95% CI)	P Value	Estimate (95% CI)	P Value
**Categories/Criteria/Characteristics**^b^	** **	** **	** **	** **	** **	** **
**Research Criteria**						
Approach	7.6 (7.5–7.7)	<0.001	7.3 (7.2–7.4)	<0.001	-	-
Significance	3.4 (3.3–3.5)	<0.001	3.4 (3.3–3.5)	<0.001	-	-
Innovation	1.4 (1.3–1.5)	<0.001	1.5 (1.4–1.5)	<0.001	-	-
Investigator(s)	1.0 (0.9–1.0)	<0.001	1.2 (1.1–1.3)	<0.001	-	-
Environment	-0.2 (-0.3– -0.1)	<0.001	-0.3(-0.4– -0.2)	<0.001	-	-
Approach X Significance	-0.8 (-0.9– -0.8)	<0.001	-0.8 (-0.9– -0.8)	<0.001	-	-
**Application Type**						
New (Type 1)	-	-	-	-	-	-
Renewal (Type 2)	-	-	-0.7 (-0.8– -0.6)	<0.001	-3.5 (-3.7– -3.3)	<0.001
Revision (Type 3)	-	-	0.5 (-0.3–1.2)	0.25	-1.3 (-2.8–0.1)	0.08
Change of Awarding IC Renewal (Type 9)	-	-	-0.7 (-1.6–0.1)	0.08	-5.0 (-6.6– -3.5)	<0.001
**Application Submission Number**						
Initial Submission (A0)	-	-	-	-	-	-
First Resubmission (A1)	-	-	-1.3 (-1.5– -1.2)	<0.001	-5.6 (-5.8– -5.4)	<0.001
Second Resubmission (A2)	-	-	-1.9 (-2.2– -1.7)	<0.001	-6.5 (-6.9– -6.1)	<0.001
**Career Stage of Investigators**						
Experienced	-	-	-	-	-	-
Early Stage Investigator (ESI)	-	-	-1.2 (-1.5– -0.8)	<0.001	1.3 (0.7–1.9)	<0.001
Non-ESI New Investigator	-	-	-0.7 (-1.0– -0.3)	<0.001	2.9 (2.3–3.5)	<0.001
**Multiple Principal Investigator (MPI) Status**						
Single PI Application	-	-	-	-	-	-
MPI Application	-	-	0.0 (-0.3–0.4)	0.78	0.5 (-0.1–1.0)	0.12
**Human and/or Animal Subject Involvement**						
No Human or Animal Subjects	-	-	-	-	-	-
Animal Subjects Only	-	-	-0.0 (-0.2–0.2)	0.99	0.2 (-0.1–0.5)	0.15
Human Subjects Only	-	-	0.2 (0.0–0.4)	<0.05	1.1 (0.7–1.4)	<0.001
Human and Animal Subjects	-	-	0.1 (-0.2–0.3)	0.54	0.5 (0.1–0.9)	<0.05
**NIH Research Grant Funding Rank of Institution**						
Rank 1–30	-	-	-	-	-	-
Rank 31–100	-	-	-0.0 (-0.1–0.1)	0.99	0.7 (0.5–0.9)	<0.001
Rank 101–200	-	-	-0.1 (-0.2–0.1)	0.45	1.2 (0.9–1.5)	<0.001
Rank > 200	-	-	0.2 (-0.0–0.4)	0.08	2.4 (2.0–2.7)	<0.001
No Previous NIH Funding	-	-	0.6 (-0.1–1.3)	0.12	4.4 (3.1–5.7)	<0.001
**Institution Type**						
Medical School	-	-	-	-	-	-
Higher Education (excl. Medical)	-	-	0.1 (-0.0–0.2)	0.07	0.1 (-0.1–0.3)	0.34
Independent Hospital	-	-	0.1 (-0.1–0.3)	0.18	-0.2 (-0.5–0.2)	0.35
Research Institute	-	-	0.1 (-0.1–0.3)	0.19	-0.7 (-1.1– -0.4)	<0.001
Other Institution	-	-	0.2 (-0.1–0.5)	0.19	-0.7 (-1.3– -0.1)	<0.05
**Race**						
White	-	-	-	-	-	-
Asian	-	-	-0.1 (-0.3–0.0)	0.07	0.4 (0.1–0.6)	<0.01
Black	-	-	0.6 (0.1–1.1)	<0.05	1.4 (0.6–2.3)	<0.01
Other Races	-	-	-0.0 (-0.3–0.2)	0.87	0.5 (0.1–0.9)	<0.05
Unknown	-	-	0.1 (-0.1–0.4)	0.33	0.9 (0.4–1.3)	<0.001
Withheld	-	-	0.3 (-0.1–0.6)	0.1	0.7 (0.1–1.3)	<0.05
**Ethnicity**						
Non-Hispanic	-	-	-	-	-	-
Hispanic	-	-	-0.1 (-0.3–0.2)	0.62	0.4 (-0.1–0.9)	0.15
MPI Multiple Ethnicities	-	-	0.2 (-0.0–0.5)	0.08	0.4 (-0.1–0.8)	0.14
Unknown	-	-	0.2 (0.1–0.3)	<0.01	0.7 (0.4–1.0)	<0.001
Withheld	-	-	0.1 (-0.2–0.4)	0.55	-0.3 (-0.9–0.3)	0.33
**Gender**						
Male	-	-	-	-	-	-
Female	-	-	-0.2 (-0.3– -0.1)	<0.001	0.4 (0.2–0.6)	<0.001
MPI Multiple Genders	-	-	-0.2 (-0.4–0.1)	0.16	-0.0 (-0.5–0.4)	0.85
Unknown	-	-	-0.4 (-1.3–0.5)	0.37	-0.1 (-1.8–1.6)	0.89
Withheld	-	-	0.4 (-0.2–1.0)	0.14	0.9 (-0.2–2.0)	0.11
**Degree**						
PhD	-	-	-	-	-	-
MD-PhD	-	-	0.3 (0.2–0.5)	<0.001	0.3 (-0.0–0.6)	0.05
MD	-	-	0.1 (-0.1–0.3)	0.25	-0.1 (-0.4–0.2)	0.45
MPI Multiple Degree Types	-	-	0.2 (-0.1–0.4)	0.21	0.3 (-0.2–0.8)	0.24
Other	-	-	1.1 (0.5–1.7)	<0.001	3.0 (1.9–4.2)	<0.001
**Age Group (Years)**						
24–35	-	-	0.1 (-0.2–0.4)	0.64	-1.0 (-1.6– -0.4)	<0.001
36–45	-	-	-	-	-	-
46–55	-	-	0.3 (0.2–0.4)	<0.001	0.7 (0.4–1.0)	<0.001
56–65	-	-	0.5 (0.3–0.6)	<0.001	0.7 (0.4–1.0)	<0.001
65+	-	-	0.7 (0.5–1.0)	<0.001	1.3 (0.9–1.8)	<0.001
MPI Multiple Age Groups	-	-	0.4 (0.1–0.7)	<0.01	0.9 (0.3–1.4)	<0.01
Unknown	-	-	0.5 (0.3–0.7)	<0.001	1.1 (0.7–1.5)	<0.001
**Fiscal Year of Application**						
2010	-	-	-	-	-	-
2011	0.3 (0.2–0.4)	<0.001	0.1 (-0.1–0.2)	0.24	-2.1 (-2.4– -1.9)	<0.001
2012	0.7 (0.5–0.8)	<0.001	0.4 (0.3–0.5)	<0.001	-3.6 (-3.9– -3.3)	<0.001
2013	1.1 (1.0–1.3)	<0.001	0.9 (0.7–1.0)	<0.001	-2.7 (-3.0– -2.4)	<0.001
**Intercept**	35.8 (35.6–36.0)	<0.001	36.1 (35.6–36.5)	<0.001	39.3 (38.4–40.1)	<0.001
**# of Applications**	71651	-	71651	-	71651	-
**Number of SRG**^c^	319	-	319	-	319	-
**R**^**2**^	0.748	-	0.757	-	0.169	-
**Intraclass Correlation**	0.042	-	0.022	-	0.024	-

^a^ Overall Impact Score Model: Criterion score coefficients represent the estimated change in the Overall Impact score due to a one point increase in the criterion score, all else equal. Application characteristic coefficients represent the estimated difference in the Overall Impact score for an application with the given characteristics compared to the baseline characteristic, all else equal.

^b^ Other application and applicant characteristics controlled for, but not shown here due to space limitations, are: Council round of review, human or animal subject concerns, solicitation type (unsolicited, program announcement or request for application), locus of review (Center for Scientific Review v. other NIH Institutes and Centers), review group type (standing study section v. special emphasis panel), direct costs requested, # of years of support requested, the NIH administering Institute or Center (IC), the geographical region of the institution and the previous NIH funding history of the applicant.

^c^ Scientific Review Groups (SRGs) include both standing study sections and special emphasis panels.

**Table 4:** Funding Model^a^ Results for R01-Equivalent Applications, FY 2010–2013.

	Sub-Model A		Sub-Model B		Sub-Model C	
	Estimate (95% CI)	P Value	Estimate (95% CI)	P Value	Estimate (95% CI)	P Value
**Categories/Criteria/Characteristics**^b^	** **	** **	** **	** **	** **	** **
**Research Criteria**						
Approach	6.2 (5.9–6.5)	<0.001	6.0 (5.7–6.3)	<0.001	-	-
Significance	2.1 (2.0–2.2)	<0.001	2.2 (2.0–2.3)	<0.001	-	-
Innovation	1.5 (1.4–1.6)	<0.001	1.5 (1.5–1.6)	<0.001	-	-
Investigator(s)	1.0 (1.0–1.1)	0.3	1.4 (1.3–1.5)	<0.001	-	-
Environment	0.9 (0.8–0.9)	<0.001	0.8 (0.8–0.9)	<0.001	-	-
Approach X Significance	1.2 (1.2–1.3)	<0.001	1.3 (1.2–1.4)	<0.001	-	-
**Application Type**						
New (Type 1)	-	-	-	-	-	-
Renewal (Type 2)	-	-	1.4 (1.3–1.5)	<0.001	2.2 (2.1–2.3)	<0.001
Revision (Type 3)	-	-	1.7 (1.2–2.6)	<0.01	2.6 (2.0–3.4)	<0.001
Change of Awarding IC Renewal (Type 9)	-	-	1.7 (1.2–2.4)	<0.01	2.9 (2.3–3.7)	<0.001
**Application Submission Number**						
Initial Submission (A0)	-	-	-	-	-	-
First Resubmission (A1)	-	-	2.2 (2.1–2.3)	<0.001	3.7 (3.6–3.8)	<0.001
Second Resubmission (A2)	-	-	2.8 (2.5–3.1)	<0.001	4.5 (4.2–4.8)	<0.001
**Career Stage of Investigators**						
Experienced	-	-	-	-	-	-
Early Stage Investigator (ESI)	-	-	2.6 (2.2–3.1)	<0.001	1.5 (1.4–1.7)	<0.001
Non-ESI New Investigator	-	-	1.7 (1.4–2.0)	<0.001	1.0 (0.9–1.1)	0.56
**Multiple Principal Investigator (MPI) Status**						
Single PI Application	-	-	-	-	-	-
MPI Application	-	-	1.0 (0.9–1.2)	0.91	1.0 (0.9–1.1)	0.64
**Human and/or Animal Subject Involvement**						
No Human or Animal Subjects	-	-	-	-	-	-
Animal Subjects Only	-	-	1.0 (1.0–1.1)	0.43	1.0 (0.9–1.0)	0.52
Human Subjects Only	-	-	0.9 (0.8–1.0)	<0.05	0.8 (0.8–0.9)	<0.001
Human and Animal Subjects	-	-	1.0 (0.8–1.1)	0.39	0.9 (0.8–0.9)	<0.001
**NIH Research Grant Funding Rank of Institution**						
Rank 1–30	-	-		-		-
Rank 31–100	-	-	1.0 (0.9–1.0)	0.33	0.8 (0.8–0.9)	<0.001
Rank 101–200	-	-	1.0 (0.9–1.1)	0.9	0.7 (0.7–0.8)	<0.001
Rank > 200	-	-	0.9 (0.8–1.0)	<0.05	0.6 (0.5–0.6)	<0.001
No Previous NIH Funding	-	-	1.0 (0.7–1.5)	0.81	0.4 (0.3–0.6)	<0.001
**Institution Type**						
Medical School	-	-	-	-	-	-
Higher Education (excl. Medical)	-	-	1.0 (0.9–1.0)	0.14	1.0 (1.0–1.0)	0.85
Independent Hospital	-	-	1.0 (0.9–1.2)	0.38	1.1 (1.0–1.2)	<0.05
Research Institute	-	-	1.0 (0.9–1.1)	0.59	1.2 (1.1–1.2)	<0.001
Other Institution	-	-	0.9 (0.8–1.1)	0.47	1.1 (1.0–1.3)	<0.05
**Race**						
White	-	-	-	-	-	-
Asian	-	-	0.9 (0.9–1.0)	0.1	0.9 (0.8–0.9)	<0.001
Black	-	-	1.0 (0.7–1.2)	0.73	0.7 (0.6–0.8)	<0.001
Other Races	-	-	1.0 (0.9–1.1)	0.95	0.9 (0.8–0.9)	<0.001
Unknown	-	-	0.9 (0.8–1.0)	0.07	0.8 (0.7–0.9)	<0.001
Withheld	-	-	0.9 (0.7–1.0)	0.09	0.9 (0.8–1.0)	<0.05
**Ethnicity**						
Non-Hispanic	-	-	-	-	-	-
Hispanic	-	-	1.0 (0.8–1.1)	0.74	0.9 (0.8–1.0)	<0.05
MPI Multiple Ethnicities	-	-	1.0 (0.9–1.1)	0.79	1.0 (0.9–1.0)	0.31
Unknown	-	-	0.9 (0.8–1.0)	<0.01	0.8 (0.8–0.9)	<0.001
Withheld	-	-	1.0 (0.9–1.2)	0.78	1.1 (0.9–1.2)	0.33
**Gender**						
Male	-	-	-	-	-	-
Female	-	-	1.0 (1.0–1.1)	0.22	0.9 (0.9–0.9)	<0.001
MPI Multiple Genders	-	-	1.0 (0.9–1.2)	0.77	1.0 (0.9–1.1)	0.46
Unknown	-	-	1.3 (0.8–2.2)	0.33	1.2 (0.8–1.7)	0.33
Withheld	-	-	0.8 (0.6–1.1)	0.22	0.9 (0.7–1.1)	0.26
**Degree**						
PhD	-	-	-	-	-	-
MD-PhD	-	-	1.0 (0.9–1.1)	0.74	1.0 (1.0–1.1)	0.27
MD	-	-	1.0 (0.9–1.1)	0.89	1.1 (1.0–1.1)	<0.05
MPI Multiple Degree Types	-	-	0.9 (0.8–1.1)	0.36	1.0 (0.9–1.0)	0.32
Other	-	-	0.5 (0.3–0.7)	<0.001	0.4 (0.3–0.5)	<0.001
**Age Group (Years)**						
24–35	-	-	0.8 (0.7–1.0)	<0.05	1.1 (1.0–1.2)	0.18
36–45	-	-	-	-	-	-
46–55	-	-	0.9 (0.8–0.9)	<0.001	0.9 (0.8–0.9)	<0.001
56–65	-	-	0.9 (0.8–0.9)	<0.001	0.9 (0.8–0.9)	<0.001
65+	-	-	0.8 (0.7–0.9)	<0.001	0.7 (0.7–0.8)	<0.001
MPI Multiple Age Groups	-	-	0.9 (0.8–1.0)	0.08	0.8 (0.8–0.9)	<0.01
Unknown	-	-	0.8 (0.7–0.9)	<0.001	0.8 (0.7–0.8)	<0.001
**Fiscal Year of Application**						
2010	-	-	-	-	-	-
2011	0.5 (0.5–0.5)	<0.001	0.5 (0.5–0.5)	<0.001	0.9 (0.9–1.0)	<0.01
2012	0.3 (0.3–0.4)	<0.001	0.4 (0.3–0.4)	<0.001	1.0 (0.9–1.0)	<0.05
2013	0.3 (0.3–0.3)	<0.001	0.3 (0.3–0.3)	<0.001	0.9 (0.8–0.9)	<0.001
**Intercept**	0.1 (0.1–0.1)	<0.001	0.0 (0.0–0.0)	<0.001	0.1 (0.1–0.1)	<0.001
**# of Applications**	111533	-	111533	-	111533	-
**Number of SRGs**^c^	318	-	318	-	318	-
**Log Likelihood**	-25242	-	-23283	-	-48290	-
**Intraclass Correlation**	0.178	-	0.14	-	0.013	-

^a^ Funding Model: (Odds Ratios) For ease of interpretation, the criterion score coefficients were inverted. Therefore, in contrast to the Impact Model, criterion score coefficients represent the estimated change in relative odds of funding due to a one point improvement (or decrease) in the criterion score, all else equal. Application characteristic coefficients represent the estimated difference in relative odds of funding for an application with the given characteristics compared to the baseline characteristic, all else equal.

^b^ Other application and applicant characteristics controlled for, but not shown here due to space limitations, are: Council round of review, human or animal subject concerns, solicitation type (unsolicited, program announcement or request for application), locus of review (Center for Scientific Review v. other NIH Institutes and Centers), review group type (standing study section v. special emphasis panel), direct costs requested, # of years of support requested, the NIH administering Institute or Center (IC), the geographical region of the institution and the previous NIH funding history of the applicant.

^c^ Scientific Review Groups (SRGs) include both standing study sections and special emphasis panels.

In sub-model B, which adds the full set of application and applicant controls to sub-model A, the coefficients of the criterion scores were largely unchanged. For the Funding model, the only major departure from sub-model A was that the Investigator(s) odds ratio coefficient increased to 1.4 (1.3–1.5), showing that applications with better Investigator(s) criterion scores were associated with better odds of funding once the other application and applicant characteristics were taken into account. Many of the application control factors had statistically significant relationships to the Overall Impact score and odds of funding. Of note, renewal applications were predicted to have Overall Impact scores 0.7 (-0.8–-0.6) points lower (better) than otherwise identical new applications and their odds of funding were predicted to be 1.4 (1.3–1.5) times better. First resubmission applications (A1s) were predicted to have Overall Impact scores 1.3 (-1.5–-1.2) points lower and odds of funding 2.2 (2.1–2.3) times greater than otherwise identical initial submissions (A0s). Applications submitted by ESIs were predicted to have Overall Impact scores 1.2 (-1.5–-0.8) points lower and odds of funding 2.6 (2.2–3.1) times greater than otherwise identical applications from experienced investigators. Applications submitted by black PIs had Overall Impact scores 0.6 (0.1–1.1) points higher or worse than applications submitted by white PIs with the same measured characteristics, though there was no statistically significant difference in odds of funding. Applications submitted by female PIs had slightly better Overall Impact scores (0.2 [-0.3–-0.1] points lower) than those submitted by male PIs, but the odds of funding were not statistically different, all else equal. See Tables 3 and 4 for the full set of control variables. Sub-model B improved the model fit and predictive accuracy of sub-model A by a very small amount, approximately one percentage point in each case.

Differences amongst subgroups in the application and applicant control variables increased substantially in sub-model C, which omits the criterion scores from the full model, sub-model B. Renewal applications were predicted to have Overall Impact scores 3.5 (-3.7–-3.3) points lower and odds of funding 2.2 (2.1–2.3) times greater than new ones. First resubmission applications were predicted to have Overall Impact scores 5.6 (-5.8–-5.4) points lower and odds of funding 3.7 (3.6–3.8) times greater than initial submissions. In contrast to sub-model B, applications submitted by ESI’s were predicted to have Overall Impact scores 1.3 (0.7–1.9) points higher or worse than experienced applications and their funding advantage was reduced to an odds ratio of 1.5 (1.4–1.7). Therefore, the ESI advantage in Overall Impact scores and funding odds was observed only after controlling for the criterion scores. Applications submitted by black PIs and female PIs appeared less likely to be funded, with the odds ratios of black PIs and female PIs falling to 0.7 (0.6–0.8) and 0.9 (0.9–0.9), respectively, and becoming statistically significant in absence of the criterion scores. The amount of variation explained by sub-model C was low (R^2^ = 16.9%) and the overall correct prediction rate was lower, 80.7% (only 9.6% for funded applications and 97.7% for unfunded applications).

## Discussion

The Impact and Funding model results demonstrate that the criterion scores are the best predictors of an application’s Overall Impact score and its likelihood of receiving funding. The model fit statistics support this observation. The R^2^, or variation explained, and correct prediction rate only improved by one percentage point when going from models which included only the criterion scores, to those which included all the other application and applicant control factors. Furthermore, when the criterion scores were removed from the full model, the variation explained and correct prediction rate fell off markedly, and the control variables increased in magnitude and many became statistically significant. Among the criterion scores, there was a clear hierarchy in terms of each criterion’s relationship with the Overall Impact score and funding odds. In both the Impact model (which contained only discussed applications) and the Funding model (which contained both discussed and non-discussed applications), the Approach score had the strongest association, with more than double the effect of the next largest predictor, the Significance score. The predictive effect of the Environment score was very small and went in a counterintuitive direction, with better Environment scores having worse Overall Impact scores and funding odds, all else equal. This finding suggests that some applications with poor Overall Impact scores can be associated with strong Environment scores, even after controlling for the other criterion scores. Furthermore, in another set of models (not shown here) where whether an application was discussed or not served as the dependent variable, the criterion score coefficients followed the same rank order, with Approach being by far the largest predictor of whether or not an application was discussed.

The criterion scores were moderately to strongly correlated with one another. This is because highly meritorious applications tended to score well on all five criteria, and vice versa for less meritorious applications. As in Lindner et al. [15], these relatively high correlations raised concerns of multicollinearity (MC). MC does not cause bias when estimating coefficients in a correctly specified model, but it can increase the variability of the estimates [19]. This problem was mitigated by the large number of applications in the model [20], which decreased the variance inflation factor (VIF) of each research criterion. VIF measures how much the variance of an estimated regression coefficient is increased because of collinearity with the other independent variables. The literature on MC typically points to VIF scores of more than 4 as potential signs of multicollinearity problems, though this is only a rule of thumb [21]. No VIF score for the criterion scores was above 2.2 in any of the models.

The summary statistics revealed relatively large differences in Overall Impact scores and funding outcomes between applications with different characteristics, such as the difference between funding rates for new and renewal applications. Sub-model C, which controlled for different application characteristics simultaneously, still exhibited these large differences. However, the multivariate models which took into account the application’s criterion scores explained many of the apparent differences in outcomes among different sorts of applications. One notable exception is the fact that ESI applications (and to a lesser extent other applications submitted by New Investigators) had a small advantage in the Impact model and a large advantage in the Funding model. This finding is reflective of NIH policy which strives to support new investigators on new R01-equivalent awards at success rates comparable to that of established investigators submitting new applications.

Consistent with the findings of Ginther et al. [11], the present study found large differences in NIH R01 funding rates by race in the absence of the measured influence of criterion scores. Criterion scores were introduced in FY 2010, and thus were not available for the applications evaluated by Ginther. Differences in outcomes by gender were also discovered in the summary data of the present study. These demographic differences diminished or disappeared once the criterion scores were included in the full models. However, bias cannot be ruled out, particularly in the first stage of peer review, where small but statistically significant differences remain in the Impact model. To ensure fairness, NIH is undertaking an extensive review of potential bias in the peer review system (see http://acd.od.nih.gov/prsub.htm). In contrast to the Impact model, the Funding model showed almost no differences in funding outcomes by demographics once all the measured characteristics of the application were taken into account.

## Conclusion

The research criterion scores, specifically the Approach and, to a lesser extent, the Significance score, are the most important predictors of an R01 application’s Overall Impact score and its likelihood of being funded. Other factors, such as the New Investigator status of the application, are associated, particularly with funding outcomes. But the model results show that the quality of the application, as measured by the criterion scores, is the best predictor of an application’s eventual success. Applicants might consider these findings when submitting future R01 applications to NIH.

## Supporting Information

S1 Fig Box Plot Distributions of Criterion Scores for Discussed R01 Applications, FY 2010–2013. S1 Fig shows the box plot distributions of the five research criterion scores (scale: 1–9) for discussed applications. Box plot whiskers extend to the most extreme data point which is no more than 1.5 times the interquartile range from the box. N = 71,651 applications.
(TIFF)

S2 Fig Box Plot Distributions of Criterion Scores for Non-Discussed R01 Applications, FY 2010–2013. S2 Fig shows the box plot distributions of the five research criterion scores (scale: 1–9) for non-discussed applications. Box plot whiskers extend to the most extreme data point which is no more than 1.5 times the interquartile range from the box. N = 52,056 applications.
(TIFF)

S1 File Impact Model Public Use Data Set. S1 File contains data on the main variables discussed at length in this paper for the 123,707 R01 applications that NIH received between FY 2010 and FY 2013.
(XLSX)

S1 Table Summary Statistics for Discussed R01-Equivalent Applications, FY 2010–2013. (DOCX)

S2 Table Summary Statistics for Non-Discussed R01-Equivalent Applications, FY 2010–2013. (DOCX)
