# Supplementary figures and images for: How Criterion Scores Predict the Overall Impact Score and Funding Outcomes for National Institutes of Health Peer-Reviewed Applications
Source: PLoS One. 2016 Jun 1;11(6):e0155060. doi: 10.1371/journal.pone.0155060 (PMC4889138; doi:10.1371/journal.pone.0155060)

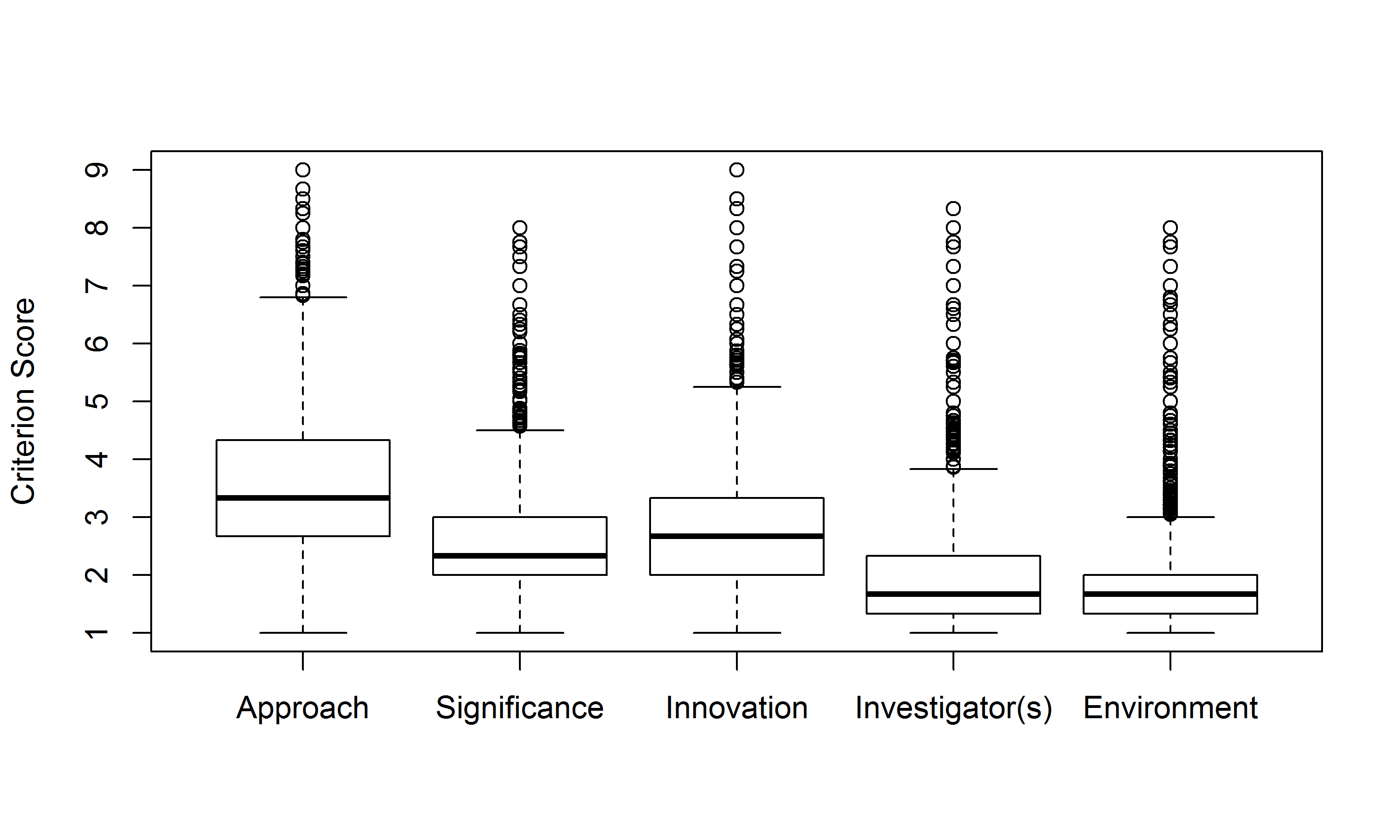

Supplement: S1 Fig — shows the box plot distributions of the five research criterion scores (scale: 1–9) for discussed applications. Box plot whiskers extend to the most extreme data point which is no more than 1.5 times the interquartile range from the box. N = 71,651 applications. (TIFF) [file pone.0155060.s001.tiff]

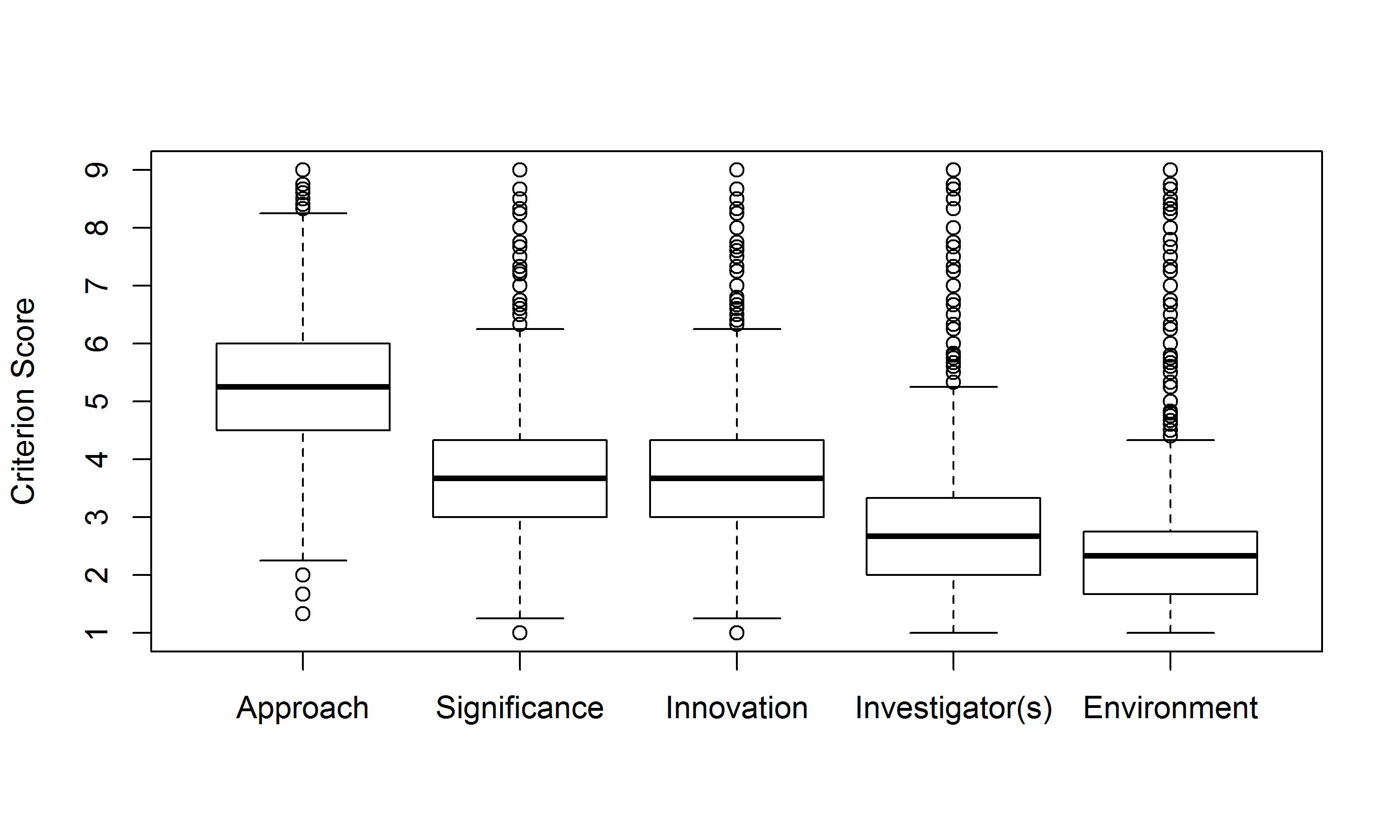

Supplement: S2 Fig — shows the box plot distributions of the five research criterion scores (scale: 1–9) for non-discussed applications. Box plot whiskers extend to the most extreme data point which is no more than 1.5 times the interquartile range from the box. N = 52,056 applications. (TIFF) [file pone.0155060.s002.tiff]
