# Supplementary material for: How Criterion Scores Predict the Overall Impact Score and Funding Outcomes for National Institutes of Health Peer-Reviewed Applications
Source: PLoS One. 2016 Jun 1;11(6):e0155060. doi: 10.1371/journal.pone.0155060 (PMC4889138; doi:10.1371/journal.pone.0155060)
Supplement: S2 Table — (DOCX) [file pone.0155060.s005.docx]

**S2 Table. Summary Statistics for Non-Discussed^a^ R01-Equivalent Applications, FY 2010-2013.**

|  | **No. (%) of Non-Discussed Applications** | **Approach Mean (st. dev)** | **Significance Mean (st. dev)** | **Innovation Mean (st. dev)** | **Investigator(s) Mean (st. dev)** | **Environment Mean (st. dev)** |
| --- | --- | --- | --- | --- | --- | --- |
| **Application/Applicant Characteristic^b^** |  |  |  |  |  |  |
| **(n=total applications, discussed and non-discussed)** |  |  |  |  |  |  |
| **Application Type^c^** |  |  |  |  |  |  |
| New (Type 1) (n=100,104) | 45,689 (45.6) | 5.3 (1.1) | 3.8 (1.1) | 3.8 (1.1) | 2.8 (1.1) | 2.4 (1) |
| Renewal (Type 2) (n=22,714) | 6,155 (27.1) | 4.8 (1) | 3.5 (1) | 3.6 (1) | 2.4 (0.9) | 2.1 (0.7) |
| Revision (Type 3) (n=568) | 128 (22.5) | 4.7 (1.1) | 3.6 (1) | 3.6 (1) | 2.3 (0.8) | 2 (0.7) |
| Change of Awarding IC Renewal (Type 9) (n=321) | 84 (26.2) | 4.8 (1) | 3.7 (1.1) | 3.5 (1) | 2.6 (1) | 2.2 (0.7) |
| **Application Submission Number^d^** |  |  |  |  |  |  |
| Initial submission (A0) (n=86,375) | 42,408 (49.1) | 5.3 (1.1) | 3.8 (1.1) | 3.8 (1.1) | 2.8 (1.1) | 2.4 (1) |
| First Resubmission (A1) (n=32,320) | 8,539 (26.4) | 5 (1.1) | 3.7 (1) | 3.7 (1) | 2.6 (1) | 2.2 (0.9) |
| Second resubmission (A2) (n=5,012) | 1,109 (22.1) | 5.1 (1) | 3.9 (1) | 3.9 (1) | 2.9 (1) | 2.5 (0.8) |
| **Career Stage of Investigators^e^** |  |  |  |  |  |  |
| Experienced Investigator (n=84,647) | 34,845 (41.2) | 5 (1) | 3.7 (1) | 3.6 (1) | 2.5 (0.9) | 2.2 (0.8) |
| Early Stage Investigator (ESI) (n=18,318) | 7,075 (38.6) | 5.5 (1) | 4 (1.1) | 4 (1.1) | 3.2 (1.1) | 2.6 (1.1) |
| Non-ESI New Investigator (n=20,742) | 10,136 (48.9) | 5.8 (1.1) | 4.2 (1.2) | 4.2 (1.2) | 3.4 (1.2) | 2.9 (1.2) |
| **Multiple Principal Investigator (MPI) Status^f^** |  |  |  |  |  |  |
| Single PI Application (n=105,235) | 44,022 (41.8) | 5.3 (1.1) | 3.8 (1.1) | 3.8 (1.1) | 2.8 (1.1) | 2.4 (1) |
| MPI Application (n=18,472) | 8,034 (43.5) | 5.1 (1.1) | 3.7 (1.1) | 3.7 (1.1) | 2.6 (1) | 2.2 (0.9) |
| **Human and/or Animal Subject Involvement^g^** |  |  |  |  |  |  |
| No Human or Animal Subjects (n=21,532) | 9,141 (42.5) | 5.2 (1.2) | 4 (1.2) | 4 (1.2) | 2.8 (1.1) | 2.5 (1) |
| Animals Subjects Only (n=55,055) | 23,256 (42.2) | 5.3 (1) | 3.8 (1) | 3.7 (1.1) | 2.8 (1) | 2.4 (0.9) |
| Humans Subjects Only (n=36,011) | 15,021 (41.7) | 5.2 (1.2) | 3.8 (1.1) | 3.8 (1.2) | 2.7 (1.2) | 2.3 (1.1) |
| Human and Animal Subjects (n=11,109) | 4,638 (41.7) | 5.2 (1) | 3.6 (1) | 3.6 (1) | 2.8 (1) | 2.3 (0.9) |
| **NIH Research Grant Funding Rank of Institution^h^** |  |  |  |  |  |  |
| Rank 1-30 (n=44,218) | 16,128 (36.5) | 5.1 (1) | 3.6 (1) | 3.6 (1.1) | 2.6 (1) | 2.1 (0.8) |
| Rank 31-100 (n=42,276) | 17,791 (42.1) | 5.2 (1.1) | 3.8 (1.1) | 3.8 (1.1) | 2.8 (1) | 2.3 (0.8) |
| Rank 101-200 (n=19,711) | 8,959 (45.5) | 5.3 (1.1) | 3.9 (1.1) | 3.8 (1.1) | 2.8 (1) | 2.5 (0.9) |
| Rank > 200 (n=16,300) | 8,364 (51.3) | 5.4 (1.1) | 4 (1.2) | 4 (1.2) | 3 (1.1) | 2.7 (1.1) |
| No Previous Funding (n=1,202) | 814 (67.7) | 6.6 (1.4) | 4.9 (1.6) | 5.1 (1.7) | 4.6 (2) | 4.4 (2) |
| **Institution Type^i^** |  |  |  |  |  |  |
| Medical School (n=64,270) | 26,258 (40.9) | 5.2 (1) | 3.7 (1) | 3.7 (1.1) | 2.8 (1) | 2.3 (0.8) |
| Higher Education (excl. Medical) (n=36,821) | 16,272 (44.2) | 5.3 (1.1) | 3.9 (1.1) | 3.9 (1.1) | 2.8 (1.1) | 2.5 (1) |
| Independent Hospital (n=9,214) | 3,610 (39.2) | 5.2 (1.1) | 3.7 (1) | 3.7 (1) | 2.7 (1) | 2.2 (0.9) |
| Research Institute (n=9,716) | 4,089 (42.1) | 5.2 (1.1) | 3.7 (1.1) | 3.8 (1.1) | 2.8 (1.1) | 2.3 (0.9) |
| Other Institution (n=3,686) | 1,827 (49.6) | 5.7 (1.4) | 4.1 (1.4) | 4.3 (1.5) | 3.4 (1.7) | 3.1 (1.8) |
| **Race^j^** |  |  |  |  |  |  |
| White (n=76,924) | 30,310 (39.4) | 5.2 (1.1) | 3.7 (1.1) | 3.7 (1.1) | 2.7 (1) | 2.3 (0.9) |
| Asian (n=24,316) | 10,987 (45.2) | 5.3 (1.1) | 3.9 (1.1) | 3.9 (1.1) | 2.9 (1) | 2.5 (0.9) |
| Black (n=1,596) | 861 (53.9) | 5.7 (1.1) | 4 (1.2) | 4.1 (1.3) | 3.2 (1.3) | 2.8 (1.3) |
| Other (n=10,014) | 4,650 (46.4) | 5.2 (1.1) | 3.7 (1.1) | 3.8 (1.1) | 2.8 (1.1) | 2.3 (1) |
| Unknown (n=7,285) | 3,744 (51.4) | 5.7 (1.2) | 4.1 (1.3) | 4.2 (1.3) | 3.4 (1.4) | 2.9 (1.4) |
| Withheld (n=3,572) | 1,504 (42.1) | 5.3 (1.1) | 3.8 (1.1) | 3.8 (1.1) | 2.9 (1.1) | 2.4 (0.9) |
| **Ethnicity^k^** |  |  |  |  |  |  |
| Non-Hispanic (n=84,563) | 34,303 (40.6) | 5.2 (1.1) | 3.8 (1.1) | 3.8 (1.1) | 2.7 (1) | 2.3 (0.9) |
| Hispanic (n=3,903) | 1,709 (43.8) | 5.3 (1.1) | 3.8 (1.1) | 3.8 (1.1) | 2.9 (1.1) | 2.5 (1) |
| MPI Multiple Ethnicities (n=8,977) | 4,006 (44.6) | 5.2 (1.1) | 3.7 (1.1) | 3.7 (1.1) | 2.7 (1.1) | 2.3 (1) |
| Unknown (n=22,514) | 10,482 (46.6) | 5.4 (1.2) | 3.9 (1.2) | 3.9 (1.2) | 3 (1.2) | 2.6 (1.1) |
| Withheld (n=3,750) | 1,556 (41.5) | 5.3 (1.1) | 3.8 (1.1) | 3.8 (1.1) | 2.8 (1.1) | 2.4 (0.9) |
| **Gender^l^** |  |  |  |  |  |  |
| Male (n=82,257) | 34,153 (41.5) | 5.2 (1.1) | 3.8 (1.1) | 3.8 (1.1) | 2.7 (1) | 2.4 (0.9) |
| Female (n=31,667) | 13,398 (42.3) | 5.3 (1.1) | 3.8 (1.1) | 3.8 (1.1) | 2.9 (1.1) | 2.4 (1) |
| MPI Multiple Gender (n=8,357) | 3,743 (44.8) | 5.2 (1.1) | 3.7 (1.1) | 3.7 (1.1) | 2.7 (1.1) | 2.3 (1) |
| Unknown (n=530) | 328 (61.9) | 6.2 (1.4) | 4.6 (1.5) | 4.7 (1.5) | 4 (1.7) | 3.6 (1.8) |
| Withheld (n=896) | 434 (48.4) | 5.6 (1.2) | 4.1 (1.2) | 4.2 (1.3) | 3.4 (1.4) | 2.9 (1.4) |
| **Degree^m^** |  |  |  |  |  |  |
| PhD (n=84,297) | 35,912 (42.6) | 5.2 (1.1) | 3.8 (1.1) | 3.8 (1.1) | 2.8 (1) | 2.4 (0.9) |
| MD-PhD (n=13,368) | 5,420 (40.5) | 5.3 (1.1) | 3.8 (1) | 3.7 (1.1) | 2.7 (1) | 2.3 (0.9) |
| MD (n=15,929) | 5,994 (37.6) | 5.2 (1.1) | 3.6 (1) | 3.7 (1.1) | 2.7 (1.1) | 2.2 (0.9) |
| MPI Multiple Degree Types (n=8,695) | 3,803 (43.7) | 5.2 (1.1) | 3.7 (1.1) | 3.7 (1.1) | 2.7 (1.1) | 2.3 (0.9) |
| Other (n=1,418) | 927 (65.4) | 6.1 (1.4) | 4.6 (1.5) | 4.7 (1.6) | 4.1 (1.7) | 3.5 (1.8) |
| **Age Group (Years)^n^** |  |  |  |  |  |  |
| 24-35 (n=3,159) | 1,281 (40.6) | 5.5 (1.1) | 4 (1.1) | 3.9 (1.2) | 3.1 (1.2) | 2.5 (1.1) |
| 36-45 (n=31,995) | 12,810 (40.0) | 5.3 (1.1) | 3.8 (1.1) | 3.8 (1.1) | 2.9 (1) | 2.4 (1) |
| 46-55 (n=36,695) | 15,377 (41.9) | 5.2 (1.1) | 3.8 (1.1) | 3.8 (1.1) | 2.8 (1) | 2.4 (0.9) |
| 56-65 (n=21,635) | 9,028 (41.7) | 5.2 (1.1) | 3.8 (1.1) | 3.8 (1.1) | 2.6 (1.1) | 2.3 (0.9) |
| 65+ (n=6,446) | 2,917 (45.3) | 5.2 (1.2) | 3.8 (1.1) | 3.8 (1.2) | 2.4 (1) | 2.2 (1) |
| MPI Multiple Age Groups (n=13,822) | 6,040 (43.7) | 5.1 (1.1) | 3.7 (1.1) | 3.7 (1.1) | 2.6 (1) | 2.2 (0.9) |
| Unknown (n=9,955) | 4,603 (46.2) | 5.4 (1.2) | 3.9 (1.2) | 3.9 (1.2) | 3 (1.2) | 2.6 (1.2) |
| **Fiscal Year of Application** |  |  |  |  |  |  |
| 2010 (n=30,487) | 12,244 (40.2) | 5.6 (1.1) | 4.1 (1.2) | 4.2 (1.2) | 3.2 (1.2) | 2.8 (1.1) |
| 2011 (n=31,216) | 13,039 (41.8) | 5.3 (1.1) | 3.8 (1.1) | 3.8 (1.1) | 2.8 (1) | 2.4 (0.9) |
| 2012 (n=31,709) | 13,644 (43.0) | 5.1 (1.1) | 3.6 (1) | 3.6 (1) | 2.6 (1) | 2.2 (0.9) |
| 2013 (n=30,295) | 13,129 (43.3) | 5.1 (1) | 3.6 (1) | 3.6 (1) | 2.6 (1) | 2.2 (0.9) |
| **Total (n=123,707)** | **52,056 (42.1)** | **5.3 (1.1)** | **3.8 (1.1)** | **3.8 (1.1)** | **2.8 (1.1)** | **2.4 (1)** |

^a^ Non-discussed applications include only those applications that were not discussed at SRG meetings. Non-discussed applications do not receive numerical Overall Impact scores. Because only one non-discussed application out of the total was funded in FY 2010-2013, the number and percent of funded applications is not presented in this table for the various application/application characteristics.

^b^ Other application and applicant characteristics evaluated, but not shown here due to space limitations, are: Council round of review, human or animal subject concerns, solicitation type (unsolicited, program announcement or request for application), locus of review (Center for Scientific Review v. other NIH Institutes and Centers), review group type (standing study section v. special emphasis panel), direct costs requested, # of years of support requested, the NIH administering Institute or Center, the geographical region of the institution and the previous NIH funding history of the applicant. For reference, n equals the number of total applications, not the number of non-discussed applications.

^c^ A new application is a type 1 application. A type 2 application is a renewal, also known as competing continuation. A type 3 application can be a competing revision for additional support to expand the scope of study or can be a non-competing administrative supplement application for additional support to cover increased costs. A type 9 application is a renewal for which the awarding institute or center changes.

^d^ An application submitted for the first time is an A0 application or an initial submission. A previously submitted unfunded A0 application resubmitted for new funding consideration is an A1 application or a first resubmission. A previously unfunded A1 application resubmitted for new funding consideration is an A2 application or a second resubmission. The policy on resubmission in place for applications submitted during the study period, FY 2010-FY 2013, can be found at <http://grants.nih.gov/grants/guide/notice-files/NOT-OD-09-003.html>.

^e^ A new investigator is defined as a principal investigator who has not previously competed successfully as a principal investigator for a substantial independent research award. A new investigator who is within 10 years of completing his/her terminal research degree or is within 10 years of completing medical residency (or equivalent) is considered an early stage investigator. A principal investigator who is not a new investigator is an experienced investigator. A list of NIH grant activities that do not disqualify a principal investigator from being considered as a new investigator can be found at <http://grants.nih.gov/grants/new_investigators/index.htm>.

^f^ An application including only one principal investigator (PI) is a single PI application. An application including more than one principal investigator is a multiple PI (MPI) application.

^g^ An application involving (1) only human subjects for research is a humans only application, (2) only animal subjects for research is an animals only application, (3) both human and animal subjects for research is a humans and animals application, and (4) neither human nor animal subjects for research is a no humans or animals application.

^h^ An application's rank is based on the rank order of the application's submitting organization or institution with respect to the total amount of NIH research grant funding received by that organization compared to all other organizations over the five year period prior to the fiscal year of the application. The lower the rank, the higher is the previous level of funding from NIH.

^i^ The type of the institution or organization submitting the application.

^j^ Race of a principal investigator is the racial category that was self-reported by the principal investigator. Applications whose principal investigator reports more than one race category or applications with multiple principal investigators who report different race categories are included in the 'Other' category.

^k^ Ethnicity of a principal investigator is the ethnicity selection that was self-reported by the principal investigator. Applications with multiple principal investigators who report different ethnicities are included in the 'MPI Multiple Ethnicities' category.

^l^ Gender of a principal investigator is the gender selection that was self-reported by the principal investigator. Applications with multiple principal investigators who report different genders are included in the 'MPI Multiple Gender' category.

^m^ Degree represents the highest degree attained by a principal investigator. Applications with multiple principal investigators reporting more than one degree type are included in the 'MPI Multiple Degree Types' category. The "Other" degree category includes degree types such as veterinary, dental and unknown degrees.

^n^ Age of a principal investigator is calculated by subtracting the principal investigator's birth year from the application's fiscal year. Applications with multiple principal investigators who report different age group categories are included in the ‘MPI Multiple Age Groups’ category. Those with an erroneous birth date (less than 24 or greater than 90) or missing birth date are included in the 'Unknown' age category.
